# Nature Interest Scale – Development and Evaluation of a Measurement Instrument for Individual Interest in Nature

**DOI:** 10.3389/fpsyg.2021.774333

**Published:** 2021-11-29

**Authors:** Matthias Winfried Kleespies, Lena Doderer, Paul Wilhelm Dierkes, Volker Wenzel

**Affiliations:** ^1^Department of Biology, Bioscience Education and Zoo Biology, Goethe University Frankfurt, Frankfurt, Germany; ^2^Department of Biology, Bioscience Education, Goethe University Frankfurt, Frankfurt, Germany

**Keywords:** Nature Interest Scale (NIS), individual interest, interest in nature, scale development, validity, reliability, university students, special needs students

## Abstract

Interest is an important factor for successful learning that has been the subject of intensive research for decades. Although interest in nature is of great importance for environmental education, to date there is no valid and reliable measurement tool. Therefore, the purpose of this study was to develop and test a scale for interest in nature, the Nature Interest Scale (NIS). In study 1, nine items were selected based on the three dimensions of the psychological interest construct to represent interest in nature. The factor structure of this new measurement instrument, was tested using confirmatory factor analyses. The results show that the instrument represents the three dimensions of the interest construct well. In study 2 the validity (discriminant and convergent validity) as well as the reliability (internal consistency, composite reliability, test-retest reliability) of the NIS were demonstrated. In study 3, the applicability of the NIS was tested with a different target group, students with learning disabilities. The results of this factor analysis also confirm the factor structure of the scale. Thus, this study provides a valid and reliable measurement tool for individual interest in nature that can be used for future research.

## Introduction

In everyday language, “interest” is often seen as motivation to learn more about a topic. Interest has also been an important topic in pedagogy and educational research for a long time: For example, the educator Dewey recognized the importance of interest more than 100 years ago (Dewey, 1913). Since that time, research on interest has evolved significantly. In particular, the examination of the construct of interest by researchers such as Schiefele (1991), Prenzel (1992), Krapp (1999) has greatly advanced research in recent decades.

Interest in educational psychology is often described as a dynamic relationship between a person and an object of interest (Person-Object-Theory of Interest; Krapp, 1993, 1998, 2000). Such an object of interest can be, for example, a topic, an idea, an activity, an event or some other content of a person’s cognitive life space (Krapp, 2002).

Interest has been studied very intensively in the last decades and is considered a very important factor in the context of learning (Renninger and Hidi, 2011). Thus, interest correlates positively with learning and thereby promotes a deeper form of understanding (Schiefele and Schreyer, 1994). Ainley et al. (2002) demonstrated that both individual interest and text titles influence learning. Interest is also crucial for the way we process information and has a positive influence on cognitive functions (Hidi, 1990). As a result, higher interest also helps to focus on tasks and to complete them (Renninger et al., 2002). Overall, interest in a subject is a good basis for a better learning success (Ainley et al., 2002).

Especially in the school and academic context, the positive effects of interest become apparent. For instance, interest in a subject is a particularly important criterion when it comes to choosing a course. In high school, natural sciences courses in particular are more likely to be chosen by students who are also more interested in natural science (Bøe, 2012). Physics, for example, is chosen both as a course at school and as a subject of study at university primarily by people who have an intrinsic interest in the subject (Bøe and Henriksen, 2013). Similar results have been documented for mathematics (Köller et al., 2000, 2001). In a longitudinal study, Harackiewicz et al. (2002) demonstrated that university students’ interest in the introductory course was strongly correlated with subsequent course enrollment and academic major over a period of 4–7 years.

Consequently, interest is directly related to academic success. Thus, interest has been shown to correlate with performance in a subject (Krapp, 1992) and interest in a discipline generally leads to better academic results in that area (Denissen et al., 2007). A performance-enhancing effect due to interest can also be assumed (Krapp, 1992). In a meta-study that examined a total of 121 studies from 18 countries, Schiefele et al. (1992) found an average correlation value of *r* = 0.31 between interest and academic success, demonstrating the positive connection between both factors. These research findings demonstrate the importance of interest in relation to learning and learning success.

In terms of structure, two basic types of interest are usually distinguished in the current literature (Krapp, 1992; Hidi et al., 2004; Schiefele, 2012): individual or personal interest on the one hand, and situational interest on the other. Situational interest is a motivated state that results from the stimulus of a specific learning situation (Krapp, 1992). Over time, situational interest may develop into a durable and stable individual interest through repeated engagement with the object of interest. Personality-specific individual interest is characterized by a dispositional internalization of a person’s interest in an object of interest. The person-object engagement can then occur of its own accord without external triggers or support (Hidi and Anderson, 1992; Renninger and Hidi, 2002; Hidi and Renninger, 2006; Pawek, 2009; Ainley, 2017). In this study, special attention will be paid to longer-term individual interest.

Individual interest is formed of three components: An emotional, a value-related, and a cognitive component (Prenzel et al., 1986; Hidi, 2006; Ainley, 2017). The emotional component can be summarized as pleasant or perceived positive feelings that occur when engaging with the object of interest (Krapp, 2002). The cognitive component of interest is the desire to expand and develop knowledge about the object of interest and learn more about it (Pawek, 2009). The third component is value-related and expresses that a high personal importance and appreciation is attached to the object of interest (Prenzel et al., 1986; Krapp and Prenzel, 2011).

In the biology education literature numerous attempts to survey interest have been made (Rowland et al., 2019). Empirically investigating the construct of interest with adequate measurement instruments offers many opportunities for scientific research: For example, scientific theories and constructs can be empirically confirmed or rejected and educational programs can also be evaluated. However, the surveys of interest in the biology education literature are often with theoretical and methodological limitations. For example, many interest studies in biology education do not adequately define interest, do not consider the theory of interest, or only cover parts of the construct (Rowland et al., 2019).

Although there are a few general instruments for measuring interest in science topics that have been designed with great emphasis on meeting quality criteria (e.g., Romine et al., 2014; Rotgans, 2015), these instruments are usually either target group-dependent or topic-specific. As a result, validated measurement instrument for specific topics, such as interest in nature, are rare. To examine whether a validated and established measurement tool for interest in nature already exists in the literature, we searched the scientific database Education Resources Information Center (ERIC). The search keywords used were “interest in nature.” Although we found some publications that tried to empirically measure interest in nature (e.g., Sjöblom and Wolff, 2017; Palmberg et al., 2019; Ahnesjö and Danielsson, 2020), no validated instrument covering the three dimensions of the psychological construct of interest was found. In most cases, these were single item measurement instruments. This leads to a research gap in environmental education research and environmental psychology on the topic of interest in nature. Therefore, this study will develop, test, and evaluate a new measurement instrument for surveying interest in nature based on interest theory: The Nature Interest Scale (NIS). The development of a new scale is a multilevel process through which the quality of the new instrument is to be ensured (Boateng et al., 2018; Carpenter, 2018).

In study 1, appropriate items for measuring interest in nature were selected on the basis of the interest construct. The factor structure of these items was then examined with confirmatory factor analyses (CFA). In study 2, the validity and reliability of the measurement instrument were explored. The third study examined whether the factor structure observed in Study 1 is also found for another target group, namely students with special needs.

## Study 1

In Study 1, nine items were selected based on the interest construct and then the factor structure of the new measurement instrument was examined using a CFA. A CFA is a common tool used when developing an instrument to examine the dimensionality of a scale and the relationship of items to each other (Brown, 2015). The advantage over a principle component analysis (PCA) is that with a CFA, the researcher has the ability to specify the factors and structure to fit the theory of the construct (Worthington and Whittaker, 2006).

### Methods

In order to guarantee the content validity of an instrument, it must represent as accurately as possible the concept that it is intended to capture (Rusticus, 2014). Therefore, according to the three components of the construct of interest, nine appropriate items were created. The items were either directly based on the definition of interest (Prenzel et al., 1986; Hidi, 2006; Ainley, 2017) or on previous interest studies on other topics (Frey et al., 2009; Holstermann, 2009; Pawek, 2009; Wenzel, 2016). All items were adapted to the study object “interest in nature.” A complete item documentation can be found in Table 1. The three components of the interest construct [emotional (EMO), cognitive (COG), and value-related (VALUE) component] were addressed with three items each which is considered to be the minimum number per factor (Raubenheimer, 2004).

**TABLE 1:** The nine items selected based on the construct of interest.

**Abbreviation**	**Item**	**Explanation for content validity**	**Origin of the item**
EMO_1	I find it exciting to deal with nature	“Exciting” as a positive feeling	(Frey et al., 2009) I find the content really exciting
EMO_2	Learning about nature is fun for me	“Fun” as a positive feeling	(Mang et al., 2018) Learning mathematics is simply fun for me
EMO_3	When I am engaged in nature, I am very concentrated and forget everything around me	If someone is so intensively involved with a subject, the person must also associate positive feelings with it	(Mang et al., 2019) I quickly forget the time when I use digital devices
COG_1	I would like to know much more about nature	Desire to obtain more knowledge	(Wenzel, 2016) I would like to know more about some topics
COG_2	I would like to learn more about nature	Desire to obtain more knowledge	(Pawek, 2009) I would like to learn more about the experiments […]
COG_3	In my free time I often deal with topics related to nature	Wanting to spend free time on a subject implies that there is a desire to know more	(Holstermann, 2009) In my free time, I also deal with the structure of the heart
VALUE_1	I find it meaningful to be involved with nature	“Meaningful” assigns a personal value to nature	(Wenzel, 2016) I find it meaningful to be involved with animals
VALUE_2	I think it’s important to be well informed about nature	“Important” assigns a personal value to nature	(Wenzel, 2016) I think it’s important to be well informed about animals
VALUE_3	The subject of nature is important to me	“Important to me” assign a value to nature	(Holstermann, 2009) To understand the function of the heart is important to me

*The last column shows the origin and the English translation of each item. The items were adapted by the authors to the topic “interest in nature” and partly modified.*

#### Participants

A total of 688 persons (66.71% female, 31.98% male, 1.31% no answer) were surveyed. Of these, 360 were students at Goethe University in Frankfurt. The survey took place in different courses of the Faculty of Biology. The questionnaire was handed out at the beginning of the class with the request to leave the completed survey at a collection point in the lecture room at the end of the class. Participants were informed prior to the survey about the project objectives and that participation was voluntary. The remaining 328 people were surveyed using an online questionnaire. Participants were recruited through email, personal contacts, and social networks. In the introductory text of the online survey, the participants were informed about the project objectives and data protection.

#### Analysis

The CFA was performed using AMOS 27. Missing values were replaced by series means and maximum likelihood estimation was used as fitting function. Common fit indices were selected to test model goodness of fit (Boateng et al., 2018). Chi-square test (χ^2^), Chi square/degrees of freedom (χ^2^/df), Root Mean Squared Error of Approximation (RMSEA), Tucker Lewis Index (TLI), Comparative Fit Index (CFI), and Standardized Root Mean Square Residual (SRMR).

**FIGURE 1:**
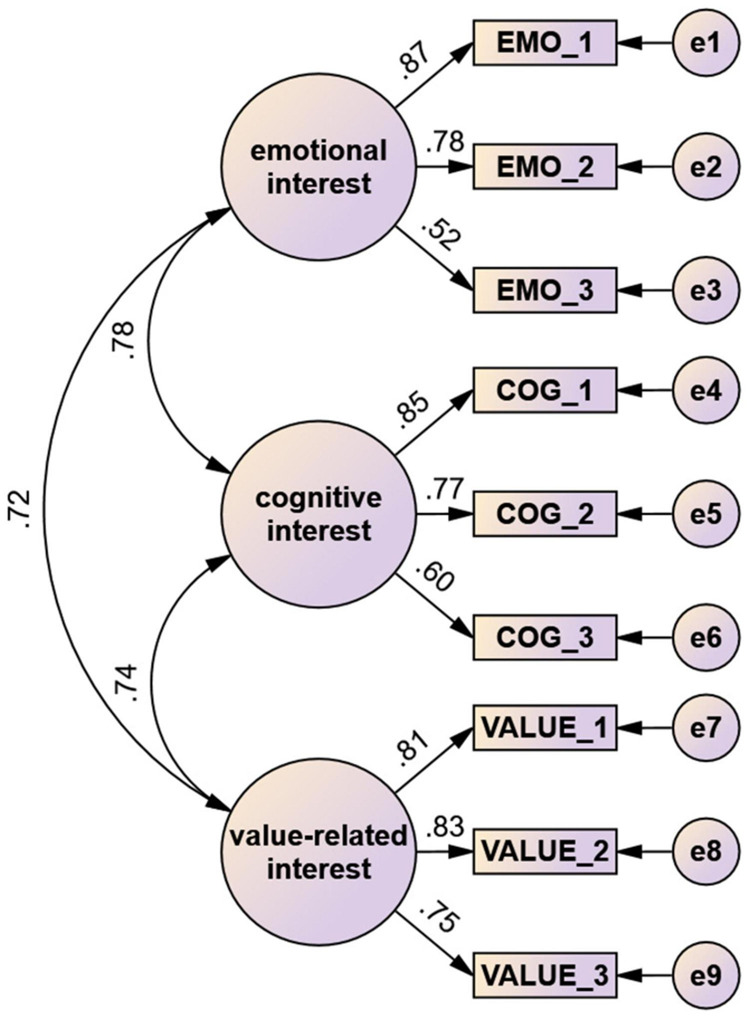
Path diagram showing the standardized results of the confirmatory factor analysis of model 2. The values between the three factors are correlations. The values between the factors and items are standardized regression weights.

Two different models were tested. In Model 1, all interest items were attributed to a single latent factor. This view is based on prior research in which interest was often treated as a unidimensional construct (Rotgans, 2015; Rowland et al., 2019). In Model 2, the interest items were assigned to three latent factors. This was based on the literature on the interest construct (Prenzel et al., 1986; Krapp, 2002; Figure 1).

### Results

The CFA revealed a high correlation between the three higher-order factors of the construct of interest (>0.7). The regression weights are also high for all items (>0.5), with an average of 0.75 (Figure 1). The fit indices differ slightly between the two models. However, Model 2 obtained slightly better values for all indices. The exact fit indices for both models can be found in Table 2.

**TABLE 2:** Results CFA for models 1 and 2.

	**χ ^2^**	***p***	**df**	**χ ^2^/df**	**CFI**	**TLI**	**SRMR**	**RMSEA**
Model 1	196.60	<0.001	27	7.282	0.952	0.935	0.034	0.096
Model 2	115.80	<0.001	24	4.825	0.974	0.961	0.032	0.075

*Both models used the same sample of 688 participants.*

### Discussion

In previous studies in which the construct of interest was measured, the factor structure of the instruments was treated differently: For example, some studies that surveyed the different aspects of the interest construct treated them as different factors (Linnenbrink-Garcia et al., 2010; Holstermann et al., 2012). In contrast, other research that also cover the different aspects of the construct treat their instruments unidimensional (Schiefele, 1990; Schiefele and Krapp, 1996; Kleespies et al., 2021). For a very thorough review on this topic, we recommend Rowland et al. (2019). To assess whether the NIS is rather unidimensional (Model 1) or multidimensional (Model 2), different fit indices were used to evaluate the models.

Since the significance of the Chi-square test depends strongly on the sample size (Marsh et al., 1988), other indices are now often used. There are no exact guidelines for the assessment of these fit indices, only rules of thumb to help in the evaluation of models. For example, the χ^2^/df ratio should be less than 5 for a sufficient model fit (Wheaton et al., 1977). Hu and Bentler (1999) recommend values close to 0.95 for the TLI and CFI, values around 0.08 for the SRMR, and values close to 0.06 for the RMSEA. However, there are also scientists who set the cutoff values slightly lower. Thus, for CFI and TLI, values greater than 0.90 may still be within the acceptable range (Browne and Cudeck, 1992; Awang, 2015), and for RMSEA values up to 0.08 or 0.10 can be tolerated (Browne and Cudeck, 1992; MacCallum et al., 1996; Awang, 2015).

Both models show a reasonable model fit. However, in a direct comparison, Model 2 shows a better model fit than Model 1. Especially the χ^2^/df and the RMSEA show differences in favor of Model 2. Therefore, the results of the CFA suggest that the NIS consists of an emotional, value-related and cognitive factor. This makes it one of the few measurement instruments in the biology education literature that is based on the theoretical construct of interest and also measures this construct multidimensionally (Rowland et al., 2019).

## Study 2

After the factor structure of the newly developed test instrument was examined in study 1, the reliability and validity of the items will be tested in study 2. To test for validity, discriminant and convergent validity were examined. To examine reliability, Cronbach’s alpha, composite reliability was calculated and test-retest reliability of the three subscales was examined.

### Methods

#### Procedure and Participants

##### Convergent and discriminant validity

To test the convergent validity of the NIS, it was compared to another interest measurement instrument, the Individual Interest Questionnaire (IIQ) by Rotgans (2015). Instead of interest in biochemistry, as in the original instrument, we asked about interest in nature. Since our survey groups were not high school students, one question was excluded in the process, as it asked about interest in school lessons on the topic. The same 360 biology students that were surveyed in study 1 served as the survey group.

To test discriminant validity, the NIS was contrasted with four questions that asked people to rate the benefits of nature to humans based on ecosystem services (Table 3). While these measurement questions are also related to nature, the assessment should not be directly related to interest in nature. A comparatively low correlation would therefore give indications of discriminant validity. The survey group used for this purpose was the people in the online survey from study 1.

**TABLE 3:** Questions for testing discriminant validity.

	**Ecosystem services (ES)**
1	Nature has a benefit because it regulates processes such as the climate, the degradation of pollutants or the pollination of plants
2	Nature has a benefit in that it provides food, water, raw materials, etc., for humans
3	Nature has value because it is responsible for processes such as recycling of nutrients, soil formation or production of biomass
4	Nature has meaning as it can serve as a place for recreation or spiritual experiences

*Since these questions do not measure a construct related to interest, there should be very little correlation between the constructs.*

##### Test-retest reliability

To determine the minimum sample size for the test-retest reliability, a power analysis with GPower (Faul et al., 2009) was performed. For a large effect (*r* = 0.7), which can be expected for test-retest reliability and a power of 0.95 (two tailed test, alpha = 0.05), there must be at least a sample size of 20 participants.

Therefore, 50 students who had taken a course in biology at the Goethe University in Frankfurt were surveyed at the beginning of the summer semester 2021 using an online questionnaire. The students were informed about the voluntary nature of their participation and the objectives of the study. Three months later, the second questionnaire was sent to the students *via* email. The time period of 3 months was chosen because the study participants should not remember the questions of the first test when taking the second test, otherwise the result would be biased. A total of 25 people participated in both surveys.

#### Analysis

All analyses were conducted using IBM SPSS 27 and Excel. To determine convergent and discriminant validity, the Pearson correlation between the NIS and the four ES items was calculated. To evaluate convergent validity, the Pearson correlation between the NIS and Rotgans (2015) IIQ scale was calculated. In addition, to test convergent validity, the Average Variance Extracted (AVE) was calculated according to Fornell and Larcker (1981):


$$AVE=\frac{\sum_{i=1}^{p}\lambda_{i}^{2}}{\sum_{i=1}^{p}\lambda_{i}^{2}+\sum_{i=1}^{p}Var\left(\varepsilon_{i}\right)}$$

To test the three subscales of the interest scale for reliability and initial consistency, in addition to the Cronbach’s Alpha, the composite reliability (CR) according to Fornell and Larcker (1981) was calculated:


$$CR=\frac{{\left(\sum_{i=1}^{p}\lambda_{i}\right)}^{2}}{{\left(\sum_{i=1}^{p}\lambda_{i}\right)}^{2}+\sum_{i=1}^{p}Var\left(\varepsilon_{i}\right)}$$

To test the test-retest reliability, the Pearson correlation between the two test time points was calculated (Vilagut, 2014).

### Results

The correlations between Rotgans (2015) IIQ scale and the three components of the NIS are high (*r*_QII–EMO_ = 0.836; *r*_QII –COG_ = 0.723; *r*_QII –VALUE_ = 0.720). The correlation between the ecosystem services and the three dimensions of interest scale are small (*r*_ES–EMO_ = 0.173; *r*_ES–COG_ = 0.136; *r*_ES–VALUE_ = 0.198). AVE, CR, and Cronbach’s alpha for the three interest subscales are shown in Table 4. The correlation between the two test time periods was *r* = 0.711 for the emotional component, *r* = 0.700 for the cognitive component, and *r* = 0.900 for the value-related component. All correlations were significant (*p* < 0.05).

**TABLE 4:** AVE, CF, and Cronbach’s alpha for the three subscales of the interest scale.

	**EMO**	**COG**	**VALUE**
AVE	0.542	0.558	0.635
CF	0.773	0.788	0.839
Cronbach’s alpha	0.792	0.737	0.840

### Discussion

In the first step, the validity of the new measurement instrument should be further examined. In addition to content validity, on which special focus was already placed in study 1 (Table 1), discriminant and convergent validity, which also belong to construct validity, are particularly important (Ginty, 2012).

An instrument is considered to have convergent validity if it has a high statistical correlation to other instruments that measure something similar. Discriminant validity, on the other hand, means that an instrument is unrelated to measurement instruments of other constructs (Campbell and Fiske, 1959). To test both types of validity the Pearson correlation can be used (Lehmann, 1988). For convergent validity, the correlation should be as high as possible so that it can be assumed that the two instruments measure the same construct (Chin and Yao, 2014). As a general guideline, the correlation for demonstrating convergent validity should be greater than *r* = 0.7 (Carlson and Herdman, 2012).

For all three factors a very high correlation (*r* > 0.7) to the IIQ scale of Rotgans (2015) could be found. This indicates that the NIS and the IIQ are very similar and presumably measure the same underlying construct (individual interest). Additionally, to verify convergent validity, the AVE was calculated. To confirm the validity of a scale, the AVE should be <0.5 (Ahmad et al., 2016). For all three subscales of the newly developed scale, the AVE is above this cutoff value. Therefore, the results confirm the presence of sufficient convergent validity.

For testing discriminant validity, there is no specific cut of value for correlation. However, the correlation should be significantly lower than for convergent validity (Hubley, 2014). All three levels of the interest scale show only a small correlation with the ES items. Because interest in nature and the ES ratings are distinct, unrelated constructs, the low correlation provides evidence of discriminant validity.

A common method to test the reliability and initial consistency of a scale is the calculation of the CR (Hatcher and O’Rourke, 2013) and the Cronbach’s alpha (Field, 2013). However, both indicators are very similar (Peterson and Kim, 2013). For the Cronbach’s alpha, values above 0.7 should be achieved (Tavakol and Dennick, 2011), for the composite reliability at least values of 0.6 (Ahmad et al., 2016). The results of both reliability measures are in an acceptable range for all three subscales. The alpha scores obtained are similar to those of other scales in the environmental field (Mayer and Frantz, 2004; Nisbet et al., 2009). Thus, reliability, internal consistency, and inter item homogeneity can be confirmed for the three subscales of the instrument.

When examining the test-retest reliability, it was found that for all three subscales a sufficiently high correlation exists between the two test times to indicate an acceptable test-retest reliability (Domino and Domino, 2006). The value-related component is shown to be particularly stable. This could be explained by the fact that the appreciation and personal valuation of nature is rather constant and almost does not change over a short period of time. It can be assumed that with a shorter survey interval (e.g., 2 weeks between the two survey dates) the test-retest reliability would have been even higher (Bühner, 2011).

## Study 3

To test whether an instrument is also suitable for a different target group, a CFA should be used (Costello and Osborne, 2005). Since the instrument should also be used with, for example, students with learning disabilities, it should be tested on a group of special needs students in study 3 and the factor structure should be re-examined.

### Methods

#### Participants

A total of 214 students (53.74% male, 44.39% female, 1.87% no answer) at three different special needs schools were surveyed. These are schools for students with learning disabilities, for whom attendance at a regular school would not be possible. Before the study was conducted approval was obtained from the relevant school authority (Hessian Ministry of Education and Religious Affairs). As part of this approval process, the consent of the school administration and the school conference was also obtained. The legal guardians of the surveyed students were informed about the study and asked for their written consent. Both parents and study participants were informed of the voluntary nature of the study. The data were collected anonymously and used for research purposes only.

Before the study began, the students were explained how a Likert scale works using everyday examples. The individual questions were read out loud to the students and a moment was waited after each question to allow the students to answer the questions. This procedure served as a supportive measure so that students with learning limitations could participate in the survey.

#### Analysis

As in study 1, a CFA was conducted to test the factor structure of the NIS for this group as well. A model was tested in which the three levels of the interest construct were represented (as in study 1 in model 2). Again, missing values were replaced by series means and maximum likelihood estimation was used as fitting function. CFI, TLI, SRMR, and RMSEA were selected as fit indices.

In the analysis of the data sets, questionnaires that showed straightlining were excluded. Straightlining refers to questionnaires in which the same answer option was selected for each question. It is conceivable, for example, that a respondent may want to express a very strong interest in nature. However, such ticking behavior can also be an expression of inattention or disinterest in the task. Since straightlining occurs particularly in younger participants (Schonlau and Toepoel, 2015), the conservative approach was chosen and these data sets (*n* = 9) were not included in the analysis.

### Results

The CFA with the data of the 205 special needs students shows a significant χ^2^ test for the performed model (*p* < 0.001). The ratio of chi-square to degree of freedom was 2.998 (χ^2^ = 71.72; df = 24). The remaining fit indices were slightly lower than the scores obtained by the university students in from study 1, with a value of 0.932 for the CFI, 0.898 for the TLI, 0.0561 for the SRMR, and 0.099 for the RMSEA.

### Discussion

The results of the CFA with the data of the special education students show a slightly worse model fit than the data of the university students. The CFI is still in the acceptable range, while the TLI is slightly below the desired cut off value of 0.90 (Browne and Cudeck, 1992). The SRMR is in the acceptable range of below 0.08 (Hu and Bentler, 1999) and the RMSEA slightly exceeds the desired value of 0.08 but is still below 0.10 (Browne and Cudeck, 1992; MacCallum et al., 1996; Awang, 2015). Since these are only indicative values and the values obtained are still within the tolerance range of some authors, it can be assumed that the model for special needs students, although not a perfect fit, still is within an acceptable range. One explanation for the slightly worse model fit could be that the young special education students do not perceive the different levels of interest as differentiated as adults. Therefore, the distinction between value-related, emotional, and cognitive interests might be less pronounced in this group. This would have the consequence that the distinction in three levels represented by the model would not be perceived as well by the special needs students as it is by adults. Nevertheless, some points speak for the usability of the scale also with special needs students. For example, the instrument is comparatively short, so that it can be completed in a relatively short time. In addition, the items are easy to understand and even students with cognitive limitations can easily comprehend what is meant by the individual items.

## Limitations

Despite the fact that the study was conducted with great care, some limitations of the research have to be considered. For example, in study 1 the scale was tested for the most part on a very homogeneous sample (university students). For future studies, it would therefore be desirable to test the scale on a more generalized group of people (e.g., other age groups or social milieus). When testing the test-retest reliability, a time period of 3 months between the two test time points was selected. It is possible that a change in interest may have occurred during this time. Interest is less stable than, for example, personal values (Feather, 1995). Nevertheless, it can be assumed that the basic interest in a topic has not changed fundamentally over a period of 3 months and thus the measurement is valid.

## Conclusion and Implications

Because of the high influence and relevance of interest, the assessment of the interest construct is still an important approach for research. However, current research often uses instruments that have not been tested for their psychological quality or do not adequately cover the construct of interest (Rowland et al., 2019). For this reason, no validated instrument for measuring interest in nature has existed in environmental psychology and environmental education research until now. This research gap shall be addressed with the NIS.

The instrument developed and tested here on interest in nature shows sufficient model fit, validity, and reliability. Thus, it offers starting points for further research. For example, it can be used to investigate the relationship between interest in nature and other environmental variables such as nature connectedness or environmental attitudes. It would be particularly useful, to compare interest in nature among individuals of different age groups. For other variables in environmental psychology such as nature connectedness or environmental attitudes, age effects have in fact already been observed (Wiernik et al., 2013; Hughes et al., 2019).

The success of environmental education programs in relation to interest in nature can also be assessed with the new measurement tool. It is already common in the evaluation of environmental education programs, for example, to look at changes in attitude, knowledge, or behavior (Braun et al., 2018). Interest in nature as a possible factor would be a useful addition here.

## Data Availability Statement

The raw data supporting the conclusions of this article will be made available by the authors, without undue reservation.

## Ethics Statement

The studies involving human participants were reviewed and approved by the Ethics Committee of the Science Didactic Institutes and Departments (FB 13, 14, 15) of the Goethe University Frankfurt am Main. Written informed consent to participate in this study was provided by the participants’ legal guardian/next of kin.

## Author Contributions

VW, PD, and MK: conceptualization, methodology, writing – review and editing, and visualization. VW, MK, and LD: data collection. MK: validation, formal analysis, investigation, and writing – original. PD: funding acquisition. VW and PD: supervision. All authors contributed to the article and approved the submitted version.

## Conflict of Interest

The authors declare that the research was conducted in the absence of any commercial or financial relationships that could be construed as a potential conflict of interest.

## Publisher’s Note

All claims expressed in this article are solely those of the authors and do not necessarily represent those of their affiliated organizations, or those of the publisher, the editors and the reviewers. Any product that may be evaluated in this article, or claim that may be made by its manufacturer, is not guaranteed or endorsed by the publisher.

## References

[B1] Ahmad S. Zulkurnain N. Khairushalimi F. (2016). Assessing the validity and reliability of a measurement model in structural equation modeling (SEM). *J. Adv. Math. Comput. Sci.* 15 1–8. 10.9734/bjmcs/2016/25183

[B2] Ahnesjö J. Danielsson T. (2020). Organized recreational fishing in school, knowledge about nature and influence on outdoor recreation habits. *J. Outdoor Environ. Educ.* 23 261–273. 10.1007/s42322-020-00061-8

[B3] Ainley M. (2017). “Interest: knowns, unknowns, and basic processes,” in *The Science of Interest*, eds O’Keefe P. A. Harackiewicz J. M. (New York, NY: Springer International Publishing), 3–24. 10.1007/978-3-319-55509-6_1

[B4] Ainley M. Hidi S. Berndorff D. (2002). Interest, learning, and the psychological processes that mediate their relationship. *J. Educ. Psychol.* 94 545–561. 10.1037/0022-0663.94.3.545

[B5] Awang Z. (2015). *SEM Made Simple: A Gentle Approach to Learning Structural Equation Modeling.* Bandar Baru Bangi: MPWS Rich Publication.

[B6] Boateng G. O. Neilands T. B. Frongillo E. A. Melgar-Quiñonez H. R. Young S. L. (2018). Best practices for developing and validating scales for health, social, and behavioral research: a primer. *Front. Public Health* 6:149. 10.3389/fpubh.2018.00149 29942800 PMC6004510

[B7] Bøe M. V. (2012). Science choices in Norwegian upper secondary school: what matters? *Sci. Educ.* 96 1–20. 10.1002/sce.20461

[B8] Bøe M. V. Henriksen E. K. (2013). Love it or leave it: Norwegian students’ motivations and expectations for postcompulsory physics. *Sci. Educ.* 97 550–573. 10.1002/sce.21068

[B9] Braun T. Cottrell R. Dierkes P. (2018). Fostering changes in attitude, knowledge and behavior: demographic variation in environmental education effects. *Environ. Educ. Res.* 24 899–920. 10.1080/13504622.2017.1343279

[B10] Brown T. A. (2015). *Confirmatory Factor Analysis for Applied Research Methodology in the Social Sciences*, 2nd Edn. New York, NY: Guilford Press.

[B11] Browne M. W. Cudeck R. (1992). Alternative ways of assessing model fit. *Sociol. Methods Res.* 21 230–258. 10.1177/0049124192021002005

[B12] Bühner M. (2011). *Einführung in Die Test- Und Fragebogenkonstruktion (3., aktualisierte und erw. Aufl.).* Munich: Pearson Studium.

[B13] Campbell D. T. Fiske D. W. (1959). Convergent and discriminant validation by the multitrait-multimethod matrix. *Psychol. Bull.* 56 81–105. 10.1037/h0046016 13634291

[B14] Carlson K. D. Herdman A. O. (2012). Understanding the impact of convergent validity on research results. *Organ. Res. Methods* 15 17–32. 10.1177/1094428110392383

[B15] Carpenter S. (2018). Ten steps in scale development and reporting: a guide for researchers. *Commun. Methods Meas.* 12 25–44. 10.1080/19312458.2017.1396583

[B16] Chin C.-L. Yao G. (2014). “Convergent validity,” in *Encyclopedia of Quality of Life and Well-Being Research*, ed. Michalos A. C. (Berlin: Springer), 1275–1276. 10.1007/978-94-007-0753-5_573

[B17] Costello A. B. Osborne J. (2005). Best practices in exploratory factor analysis: four recommendations for getting the most from your analysis. *Pract. Assess. Res. Eval.* 10 1–9. 10.7275/jyj1-4868

[B18] Denissen J. J. A. Zarrett N. R. Eccles J. S. (2007). I like to do it, I’m able, and I know I am: longitudinal couplings between domain-specific achievement, self-concept, and interest. *Child Dev.* 78 430–447. 10.1111/j.1467-8624.2007.01007.x 17381782

[B19] Dewey J. (1913). *Riverside Educational Monographs. Interest and Effort in Education.* New York, NY: Mifflin and Company.

[B20] Domino G. Domino M. L. (2006). *Psychological Testing: An Introduction*, 2nd Edn. Cambridge: Cambridge University Press.

[B21] Faul F. Erdfelder E. Buchner A. Lang A.-G. (2009). Statistical power analyses using G^∗^Power 3.1: tests for correlation and regression analyses. *Behav. Res. Methods* 41 1149–1160. 10.3758/BRM.41.4.1149 19897823

[B22] Feather N. T. (1995). Values, valences, and choice: the influences of values on the perceived attractiveness and choice of alternatives. *J. Pers. Soc. Psychol.* 68 1135–1151. 10.1037/0022-3514.68.6.1135

[B23] Field A. (2013). *Discovering Statistics Using IBM SPSS Statistics: And Sex and Drugs and Rock ‘n’ roll*, 4th Edn. Thousand Oaks, CA: Sage.

[B24] Fornell C. Larcker D. F. (1981). Evaluating structural equation models with unobservable variables and measurement error. *J. Market. Res.* 18:39. 10.2307/3151312

[B25] Frey A. Taskinen P. Schütte K. Deutschland P.-K. (2009). *PISA 2006 Skalenhandbuch: Dokumentation der Erhebungsinstrumente (1. Auflage).* Münster: Waxmann Verlag GmbH.

[B26] Ginty A. T. (2012). “Construct validity,” in *Springer Reference. Encyclopedia of Behavioral Medicine*, ed. Gellman M. D. (Berlin: Springer), 487.

[B27] Harackiewicz J. M. Barron K. E. Tauer J. M. Elliot A. J. (2002). Predicting success in college: a longitudinal study of achievement goals and ability measures as predictors of interest and performance from freshman year through graduation. *J. Educ. Psychol.* 94 562–575. 10.1037/0022-0663.94.3.562

[B28] Hatcher P. D. L. O’Rourke R. D. P. N. (2013). *Step-by-Step Approach to Using SAS for Factor Analysis and Structural Equation Modeling.* Cary, NC: SAS Institute.

[B29] Hidi S. Renninger K. A. Krapp A. (2004). “Interest, a motivational variable that combines affective and cognitive functioning,” in *The Educational Psychology Series. Motivation, Emotion, and Cognition: Integrative Perspectives on Intellectual Functioning and Development*, eds Dai D. Y. Sternberg R. J. (Mahwah, NJ: Lawrence Erlbaum Associates Publishers), 89–115.

[B30] Hidi S. (1990). Interest and its contribution as a mental resource for learning. *Rev. Educ. Res.* 60 549–571. 10.3102/00346543060004549

[B31] Hidi S. (2006). Interest: a unique motivational variable. *Educ. Res. Rev.* 1 69–82. 10.1016/j.edurev.2006.09.001

[B32] Hidi S. Anderson V. (1992). “Situational interest and its impact on reading and expository writing,” in *The Role of Interest in Learning and Development*, eds Renninger K. A. Hidi S. Krapp A. (Mahwah, NJ: Lawrence Erlbaum Associates Publishers).

[B33] Hidi S. Renninger K. A. (2006). The four-phase model of interest development. *Educ. Psychol.* 41 111–127. 10.1207/s15326985ep4102_4

[B34] Holstermann N. (2009). *Interesse Von Schülerinnen und Schülern an Biologischen Themen: Zur Bedeutung Von Hands-on Erfahrungen und Emotionalem Erleben.* Ph.D. thesis. Göttingen: Georg-August-University.

[B35] Holstermann N. Ainley M. Grube D. Roick T. Bögeholz S. (2012). The specific relationship between disgust and interest: relevance during biology class dissections and gender differences. *Learn. Instr.* 22 185–192. 10.1016/j.learninstruc.2011.10.005

[B36] Hu L. T. Bentler P. M. (1999). Cutoff criteria for fit indexes in covariance structure analysis: conventional criteria versus new alternatives. *Struct. Equ. Model.* 6 1–55. 10.1080/10705519909540118

[B37] Hubley A. M. (2014). “Discriminant validity,” in *Encyclopedia of Quality of Life and Well-Being Research*, ed. Michalos A. C. (Berlin: Springer), 1664–1667. 10.1007/978-94-007-0753-5_751

[B38] Hughes J. Rogerson M. Barton J. Bragg R. (2019). Age and connection to nature: when is engagement critical? *Front. Ecol. Environ.* 17:265–269. 10.1002/fee.2035

[B39] Kleespies M. W. Montes N. Á Bambach A. M. Gricar E. Wenzel V. Dierkes P. W. (2021). Identifying factors influencing attitudes towards species conservation–a transnational study in the context of zoos. *Environ. Educ. Res.* 27 1421–1439. 10.1080/13504622.2021.1927993

[B40] Köller O. Baumert J. Schnabel K. (2001). Does interest matter? The relationship between academic interest and achievement in mathematics. *J. Res. Math. Educ.* 32:448. 10.2307/749801

[B41] Köller O. Daniels Z. Schnabel K. U. Baumert J. (2000). Kurswahlen von Mädchen und Jungen im Fach Mathematik: zur rolle von fachspezifischem Selbstkonzept und interesse. *Z. Pädagogische Psychol.* 14 26–37. 10.1024//1010-0652.14.1.26

[B42] Krapp A. (1998). Entwicklung und Förderung von interessen im unterricht. *Psychol.Erzieh. Unterr.* 45 186–203.

[B43] Krapp A. (1992). Interesse, Lernen und Leistung. Neue Forschungsansätze in der Pädagogischen psychologie. *Z. Päd.* 5 747–770.

[B44] Krapp A. (1993). The construct of interest: characteristics of indvidual interests and interest-related actions from the perspective of a person-object-theory. *Stud. Educ. Psychol.* 4 1–18.

[B45] Krapp A. (1999). Interest, motivation and learning: an educational-psychological perspective. *Eur. J. Psychol. Educ.* 14 23–40. 10.1007/BF03173109

[B46] Krapp A. (2000). “Interest and Human development during adolescence: an educational-psychological approach,” in *Advances in Psychology. Motivational psychology of Human Development: Developing Motivation and Motivating Development*, Vol. 131 ed. Heckhausen J. (North-Holland: Elsevier), 109–128. 10.1016/S0166-4115(00)80008-4

[B47] Krapp A. (2002). “An educational-psychological theory of interest and its relation to SDT,” in *Handbook of Self-Determination Research*, eds Deci E. L. Ryan R. M. (Rochester, NY: University of Rochester Press). 10.18821/0869-2084-2018-63-5-267-272

[B48] Krapp A. Prenzel M. (2011). Research on interest in science: theories, methods, and findings. *Int. J. Sci. Educ.* 33 27–50. 10.1080/09500693.2010.518645

[B49] Lehmann D. R. (1988). An alternative procedure for assessing convergent and discriminant validity. *Appl. Psychol. Meas.* 12 411–423. 10.1177/014662168801200409

[B50] Linnenbrink-Garcia L. Durik A. M. Conley A. M. Barron K. E. Tauer J. M. Karabenick S. A. (2010). Measuring situational interest in academic domains. *Educ. Psychol. Meas.* 70 647–671. 10.1177/0013164409355699

[B51] MacCallum R. C. Browne M. W. Sugawara H. M. (1996). Power analysis and determination of sample size for covariance structure modeling. *Psychol. Methods* 1 130–149. 10.1037/1082-989X.1.2.130

[B52] Mang J. Ustjanzew N. Leßke I. Schiepe-Tiska A. Reiss K. (2019). *PISA 2015 Skalenhandbuch: Dokumentation der Erhebungsinstrumente.* Münster: Waxmann.

[B53] Mang J. Ustjanzew N. Schiepe-Tiska A. Prenzel M. Sälzer C. Müller K. (2018). *PISA 2012 Skalenhandbuch: Dokumentation der Erhebungsinstrumente.* Münster: Waxmann.

[B54] Marsh H. W. Balla J. R. McDonald R. P. (1988). Goodness-of-fit indexes in confirmatory factor analysis: the effect of sample size. *Psychol. Bull.* 103 391–410. 10.1037/0033-2909.103.3.391

[B55] Mayer F. S. Frantz C. M. (2004). The connectedness to nature scale: a measure of individuals’ feeling in community with nature. *J. Environ. Psychol.* 24 503–515. 10.1016/j.jenvp.2004.10.001

[B56] Nisbet E. K. Zelenski J. M. Murphy S. A. (2009). The nature relatedness scale: linking individuals’ connection with nature to environmental concern and behavior. *Environ. Behav.* 41 715–740. 10.1177/0013916508318748

[B57] Palmberg I. Kärkkäinen S. Jeronen E. Yli-Panula E. Persson C. (2019). Nordic student teachers’ views on the most efficient teaching and learning methods for species and species identification. *Sustainability* 11:5231. 10.3390/su11195231

[B58] Pawek C. (2009). *Schülerlabore als Interessefördernde Außerschulische Lernumgebungen für Schülerinnen und Schüler aus der Mittel- und Oberstufe.* Ph.D. thesis. Kiel: Christian-Albrecht University.

[B59] Peterson R. A. Kim Y. (2013). On the relationship between coefficient alpha and composite reliability. *J. Appl. Psychol.* 98 194–198. 10.1037/a0030767 23127213

[B60] Prenzel M. (1992). “The selective persistence of interest,” in *The Role of Interest in Learning and Development*, eds Renninger K. A. Hidi S. Krapp A. (Mahwah, NJ: Lawrence Erlbaum Associates, Inc), 71–98.

[B61] Prenzel M. Krapp A. Schiefele U. (1986). Grundzüge einer pädagogischen interessentheorie. *Z. Päd.* 32 163–173.

[B62] Raubenheimer J. (2004). An item selection procedure to maximise scale reliability and validity. *SA J. Ind. Psychol.* 30 59–64. 10.4102/sajip.v30i4.168

[B63] Renninger K. A. Hidi S. (2002). “Student interest and achievement: developmental issues raised by a case study,” in *Educational Psychology Series. Development of Achievement Motivation*, eds Wigfield A. Eccles J. (Cambridge, MA: Academic Press), 173–195. 10.1016/B978-012750053-9/50009-7

[B64] Renninger K. A. Hidi S. (2011). Revisiting the conceptualization, measurement, and generation of interest. *Educ. Psychol.* 46 168–184. 10.1080/00461520.2011.587723

[B65] Renninger K. A. Ewen L. Lasher A. K. (2002). Individual interest as context in expository text and mathematical word problems. *Learn. Instr.* 12 467–490. 10.1016/S0959-4752(01)00012-3

[B66] Romine W. Sadler T. D. Presley M. Klosterman M. L. (2014). Student interest in technology and science (SITS) survey: development, validation, and use of a new instrument. *Int. J. Sci. Math. Educ.* 12 261–283. 10.1007/s10763-013-9410-3

[B67] Rotgans J. I. (2015). Validation study of a general subject-matter interest measure: the individual interest questionnaire (IIQ). *Health Prof. Educ.* 1 67–75. 10.1016/j.hpe.2015.11.009

[B68] Rowland A. A. Knekta E. Eddy S. Corwin L. A. (2019). Defining and measuring students’ interest in biology: an analysis of the biology education literature. *CBE Life Sci. Educ.* 18:ar34. 10.1187/cbe.19-02-0037 31397650 PMC6755315

[B69] Rusticus S. (2014). “Content validity,” in *Encyclopedia of Quality of Life and Well-Being Research*, ed. Michalos A. C. (Berlin: Springer), 1261–1262. 10.1007/978-94-007-0753-5_553

[B70] Schiefele U. (1990). “The influence of topic interest, prior knowledge, and cognitive capabilities on text comprehension,” in *Recent Research in Psychology. Learning Environments: Contributions from Dutch and German Research*, ed. Pieters J. M. (Berlin: Springer), 323–338. 10.1007/978-3-642-84256-6_25

[B71] Schiefele U. Krapp A. Winteler A. (1992). “Interest as a predictor of academic achievement: a meta-analysis of research,” in *The Role of Interest in Learning and Development*, eds Renninger K. A. Hidi S. Krapp A. (Mahwah, NJ: Lawrence Erlbaum Associates, Inc.), 183–212. 10.1542/peds.2018-3556

[B72] Schiefele U. Schreyer I. (1994). Intrinsische lernmotivation und lernen. Ein überblick zu ergebnissen der forschung [Intrinsic motivation to learn and learning: a review of recent research findings]. *German J. Educ. Psychol.* 8 1–13. 10.1007/978-3-531-20002-6_40-1

[B73] Schiefele U. (1991). Interest, learning, and motivation. *Educ. Psychol.* 26 299–323. 10.1080/00461520.1991.9653136

[B74] Schiefele U. (2012). “Interests and learning,” in *Springer Reference. Encyclopedia of the Sciences of Learning: With 68 Tables*, ed. Seel N. M. (Berlin: Springer), 1623–1626. 10.1007/978-1-4419-1428-6_351

[B75] Schiefele U. Krapp A. (1996). Topic interest and free recall of expository text. *Learn. Individ. Differ.* 8 141–160. 10.1016/S1041-6080(96)90030-8

[B76] Schonlau M. Toepoel V. (2015). Straightlining in WEB survey panels over time. *Surv. Res. Methods* 9 125–137. 10.18148/SRM/2015.V9I2.6128

[B77] Sjöblom P. Wolff L.-A. (2017). “It wouldn’t be the same without nature” – the value of nature according to Finnish upper secondary school students. *J. Environ. Educ.* 48 322–333. 10.1080/00958964.2017.1367637

[B78] Tavakol M. Dennick R. (2011). Making sense of Cronbach’s alpha. *Int. J. Med. Educ.* 2 53–55. 10.5116/ijme.4dfb.8dfd 28029643 PMC4205511

[B79] Vilagut G. (2014). “Test-retest reliability,” in *Encyclopedia of Quality of Life and Well-Being Research*, ed. Michalos A. C. (Berlin: Springer), 6622–6625. 10.1007/978-94-007-0753-5_3001

[B80] Wenzel V. (2016). *Konzeption und Evaluation Eines Handlungsorientierten Lernangebotes für die Primarstufe im Außerschulischen Lernort Wildpark.* Ph.D. thesis. Frankfurt am Main: Goethe University.

[B81] Wheaton B. Muthen B. Alwin D. F. Summers G. F. (1977). Assessing reliability and stability in panel models. *Sociol. Methodol.* 8:84. 10.2307/270754

[B82] Wiernik B. M. Ones D. S. Dilchert S. (2013). Age and environmental sustainability: a meta-analysis. *J. Manag. Psychol.* 28 826–856. 10.1108/JMP-07-2013-0221

[B83] Worthington R. L. Whittaker T. A. (2006). Scale development research. *Couns. Psychol.* 34 806–838. 10.1177/0011000006288127

